# An evaluation of EQ-5D-3L health utility scores using five country-specific tariffs in a rural population aged 45–69 years in Hua county, Henan province, China

**DOI:** 10.1186/s12955-020-01476-z

**Published:** 2020-07-13

**Authors:** Hui Wang, Changqi Cao, Chuanhai Guo, Yu He, Fenglei Li, Ruiping Xu, Mengfei Liu, Zhen Liu, Yaqi Pan, Fangfang Liu, Ying Liu, Jingjing Li, Hong Cai, Zhonghu He, Yang Ke

**Affiliations:** 1 Key laboratory of Carcinogenesis and Translational Research (Ministry of Education/Beijing), Laboratory of Genetics, Peking University Cancer Hospital & Institute, Beijing, People’s Republic of China; 2 Key laboratory of Carcinogenesis and Translational Research (Ministry of Education/Beijing), Department of Endoscopy Center, Peking University Cancer Hospital & Institute, Beijing, People’s Republic of China; 3 Department of Non-communicable Disease Epidemiology, London School of Hygiene & Tropical Medicine, London, UK; 4 Hua County People’s Hospital, Anyang, Henan Province People’s Republic of China; 5 Anyang Cancer Hospital, Anyang, Henan Province People’s Republic of China

**Keywords:** EQ-5D-3L, Health utility scores, Tariffs, Rural China

## Abstract

**Background:**

This study aims to compare the performance of the recently developed Chinese (city) tariff of the EQ-5D-3L against the UK, US, Japanese and Korean tariffs in a general rural population in China.

**Methods:**

From November 2015 to September 2016, 12,085 permanent residents aged 45–69 from 257 villages randomly selected from Hua County, Henan Province, China, were interviewed using EQ-5D-3L, and a one-on-one questionnaire investigation was used to collect data on factors associated with HRQOL. The health utility scores were calculated using the UK, US, Japanese, Korean and Chinese (city) tariffs. The agreement, known-groups validity and sensitivity of these five tariffs were evaluated. Transition scores for pairs of observed EQ-5D-3L health states were calculated and compared.

**Results:**

The Korean tariff yielded the highest mean health utility score (0.963), followed by the Chinese (city) (0.948), US (0.943), UK (0.930) and Japanese (0.921) tariffs, but the differences in the scores of any two tariffs did not exceed the MCID. The Chinese (city) tariff showed higher ICC values (ICCs> 0.89, 95% CI:0.755–0.964) and narrower limits of agreement (0.099–0.167) than the Korean tariff [(ICCs> 0.71, 95% CI:0.451–0.955); (0.146–0.253)]. The Chinese (city) tariff had a higher relative efficiency and effect size statistics in 10 out of 11 variables as compared to the UK, US and Japanese tariffs. The Chinese (city) tariff (0.215) was associated with moderate mean absolute transition scores compared with the UK (0.342), US (0.230), Japanese (0.149) and Korean (0.189) tariffs for 1485 observed pairs of the EQ-5D-3L health states.

**Conclusions:**

Health utility scores derived from the five tariffs differed. The Chinese (city) tariff was the most suitable of these tariffs and was without obvious weakness. We recommend adopting the Chinese (city) tariff when applying EQ-5D-3L to assess quality of life among the elderly in China’s agricultural region with socio-economic status similar to Hua County. Results of this study had provided a crucial basis for health surveys, health promotion projects, health intervention trials, and health economic evaluation taking HRQOL as a target in rural areas of China.

## Background

Measures of health related quality of life (HRQOL) have become increasingly important in evaluating outcomes of health-care programs [1]. Among generic instruments of HRQOL, the three-level European quality of life five-dimension (EQ-5D-3L) scale is a widely applied preference-based outcome measure worldwide, as it is simple, easy to apply and yields high response rates [2]. The validity and reliability of the EQ-5D-3L scale has been tested in China [3], and has been recommended as a tool for health technology assessment in China in the publication “*China Guidelines for Pharmacoeconomic Evaluations and Manual 2015*” [2]. Its descriptive system classifies a health state into three levels (no problem, some/moderate problems, severe/extreme problems) on five dimensions: mobility, self-care, usual activities, pain/discomfort and anxiety/depression, resulting in 243 possible health states. Each health state can be converted into health utility scores with tariffs derived primarily from samples of the general public, patients or healthcare providers. Health utility scores represent an individual’s overall health status, and generally range from 0.0 (death) to 1.0 (perfect health). Some very poor health states, such as a persistent vegetative state, may be represented by health utility scores below 0.0 [4].

Evidence has shown that factors such as political systems, social culture and economic growth may affect the utility values of EQ-5D-3L health states [5, 6], so people’s evaluation on same health problems varied from different counties. For example, anxiety/depression problems had different effects on people from countries with different economic levels. The utility scores for health state “11,112” (moderate problems in anxiety/depression dimension) were 0.875 and 0.785 respectively in the perspective of Chinese urban population [7] and Japanese population [8]. Therefore, the choice of tariff may lead to different cost-utility results and affect decision-making [6]. It had commonly been suggested that adopting a population-specific tariff would better reflect the health preferences of the target population [5]. Before a China-specific tariff was available, tariffs derived from the United Kingdom (UK), the United States (US), Japan and Korea were recommended for use in China, and the Japanese and Korean tariffs were the best of these due to geographic proximity and cultural similarities [2]. Liu et al. developed Chinese utility values for EQ-5D-3L health states in 2014 based on a population-based sample drawn from developed cities including Beijing, Guangzhou, Shenyang, Nanjing and Chengdu [7]. China, however, is a traditional agricultural country. By the end of 2017, up to 42.65% of the Chinese population still lived in rural areas in the context of rapid urbanization. The Chinese central government implemented a series of health promotion projects, health surveys and health intervention trials in rural populations over the course of the 13th five-year plan [9] and it was essential to accurately measure HRQOL in rural populations for these health-care programs. At present, China has not developed a tariff from rural populations. In view of the socio-economic differences in urban and rural areas [9, 10], it is unclear whether the health preferences of urban residents can be applied to measure the HRQOL in rural populations in China, and this warrants rigorous evaluation. The previous studies on psychometric properties of HRQOL derived from the UK, US, Japanese, Korean and Chinese (city) tariffs were mainly limited to patient populations in China [11, 12], and only one study in general populations on the UK, US, Korean and Chinese (city) tariffs was conducted in urban China [13]. No specific evaluation was carried out in general rural populations in China.

In this study, we aimed to evaluate all potentially appropriate tariffs derived from the UK, US, Japan, Korea and China (city) in over 12,000 rural adults aged 45–69 on the basis of a large randomized controlled trial in rural China [14]. Findings will provide fundamental evidence for selection of an appropriate tariff for HRQOL assessment and health economic evaluation in rural China.

## Methods

### Study subjects

Hua County is an agricultural region in the northern part of Henan Province, China. It is a typical rural area with a rural population of approximately 954,000, which accounts for 68.9% of the total population of the county. The per capita Gross Domestic Product (GDP) of this area was $2627 in 2017 [9].

In January 2012, we initiated the Endoscopic Screening for Esophageal Cancer in China (ESECC) randomized controlled trial (Clinical trial: NCT01688908) in Hua County to evaluate the efficacy and cost-effectiveness of population level endoscopic screening for esophageal squamous cell carcinoma. The inclusion criteria in the ESECC trial were: 1) permanent residency in a target village; 2) age 45–69 (with > 5 years of life expectancy) and no history of endoscopic examination within 5 years prior to the initial interview; 3) no history of cancer or mental disorder; 4) negative for hepatitis B virus, hepatitis C virus and human immunodeficiency virus; 5) agreement to complete all phases of the trial. There are a total of 968 villages in rural Hua County, and 668 target villages were randomly selected from the 846 villages with population sizes ranging from 500 to 3000 in Hua County [14].

From November 2015 to September 2016, we investigated 12,085 residents aged 45–69 from 257 target villages of the ESECC trial for the current study.

### Data collection

Enrollment work was carried out in villages, and village doctors were responsible for notifying potential participants. A special computer program aided one-on-one questionnaire investigation was conducted face to face by investigators well-trained in this interview to investigate potential factors associated with HRQOL. The study questionnaire elicited background information associated with HRQOL, such as demographic factors (age, gender), socio-economic status (educational level, job type and annual household per capita income), living conditions (marital status, living arrangement), dietary habits (frequency of eating fruits and vegetables), health conditions [body mass index (BMI) and chronic diseases], followed by the EQ-5D-3L scale.

### Statistical analysis

We converted EQ-5D-3L scale responses into health utility scores using the UK [15], US [16], Japanese [8], Korean [17] and Chinese (city) [7] tariffs. One-way analysis of variance (ANOVA) with post-hoc Bonferroni tests (0.001/10) was used to examine the statistical significance of the mean derived from the five tariffs. A minimal clinically important difference (MCID) was defined as a difference of at least 0.074 in the EQ-5D-3L tariffs, and this MCID was estimated in a study which used the UK tariff and participant data from eight published longitudinal studies [18].

The level of agreement in the health utility scores derived from the UK, US, Japanese, Chinese (city) and Korean tariffs was assessed by intraclass correlations coefficients (ICCs) [19] and Bland-Altman plots [20]. The ICCs were calculated using two-way random effect models with absolute agreement to examine the extent to which these five tariffs produced the same utility scores. Agreement was considered poor for ICC values less than 0.40, fair for values between 0.40 and 0.59, good for values between 0.60 and 0.74, and excellent for values between 0.75 and 1.0 [19]. Bland-Altman plots were used to graphically depict individual-level differences in the five tariffs [20]. Bland-Altman plots showed 95% limits of agreement, which encompassed 95% of the individual-level differences in our sample. Narrow limits reflected smaller differences between any two of the five tariffs. The mean of the differences (d) and the limits of agreement (95% CI of d) were indicated by lines in the Bland-Altman plots. To assess the significance of these differences, we counted the number of participants for whom the difference exceeded the MCID.

Known-groups validity was used to evaluate whether the five tariffs had the ability to discriminate subjects from known-subgroups with different health status. Prior to estimating known-groups validity of the five tariffs, we first used the Chi-square test to examine whether the proportion of full health was significantly different in subgroups by age, gender, educational level, job type, annual household per capita income, marital status, living arrangement, frequency of eating fruits and vegetables, BMI and chronic diseases. The independent t-test was used to identify whether the mean difference in health utility scores derived from the five tariffs had the ability to discriminate all known-subgroups with different health status.

The sensitivity of these five tariffs was estimated with the relative efficiency (RE) statistic and Cohen’s d effect size (ES) statistic [21]. The RE statistic was used to compare the sensitivity of these five tariffs for discriminating among known-subgroups, which was calculated as the ratio of the Z statistic for the UK, US, Japanese and Korean tariffs divided by the Z statistic for the Chinese (city) tariff. The Mann-Whitney test was used to calculate the Z statistic. The ES statistic was computed as the differences in the mean of known-subgroups divided by the pooled standard deviation, and effect sizes of 0.2, 0.5, 0.8 indicated small, medium, and large differences respectively.

In addition, we assessed the performance of these tariffs in term of health transition scores for observed pairs of EQ-5D-3L health states. We measured the absolute mean transition score in EQ-5D-3L tariffs for observed pairs of EQ-5D-3L health states. These absolute transition scores measure the health utility gained for a transition from a worse health state to a better health state [6]. These absolute transition scores from the five tariffs were compared using one-way ANOVA with post-hoc Bonferroni tests (0.001/10). The consistency of the five tariffs in predicting positive (health gain) or negative (health loss) transition scores for observed pairs of EQ-5D-3L health states was assessed based on Chinese (city) tariff. Responsiveness of the five tariffs to these consistent health transitions was assessed using standardized response mean (SRM). SRM was calculated as the post-treatment mean minus the baseline mean and divided by the standard deviation of the changed scores between baseline and post-treatment [6]. In addition, to assess clinical importance of the differences between transition scores by different tariffs, we computed the absolute mean differences of transition scores between China (city) and four other tariffs and compared them with the MCID of EQ-5D-3L.

As a result of rigorous logical checking of our system of investigation, there were few missing data in the variables from the questionnaire. The annual household per capita income, living arrangement and BMI variables were not available for 1002 (8.29%), 3 (0.02%) and 41 (0.34%) subjects respectively, and these subjects were excluded from analysis.

All statistical analyses were performed using STATA version 15.0 (STATA Corporation, College Station, TX, USA). All tests were two-sided and had a significance level of 0.001.

## Results

From 2015 to 2016, a total of 12,085 permanent residents from rural Hua County were enrolled in this study. As shown in Table 1, 30.62% of the participants reported health problems, and lower health status was associated with older age, female gender, lower levels of education, working a labor job, lower annual household per capita income, being unmarried, living alone, low intake of fruits and vegetables, obesity, and affliction with chronic disease.

**Table 1:** Selected demographic and behavioral characteristics in 12,085 residents from rural Hua County, China

**Variable**	**N (%)**	**Full health **^**a**^**(%)**	***p*value**^**b**^
**Age (years)**			
45–54	5891 (48.75)	71.46	< 0.001
55–69	6194 (51.25)	67.39	
**Gender**			
Male	5913 (48.93)	73.33	< 0.001
Female	6172 (51.07)	65.59	
**Educational level**			
Middle School and above (>6y)	5610 (46.42)	71.76	< 0.001
Primary School and below (≤6y)	6475 (53.58)	67.31	
**Job type**			
Office	214 (1.77)	75.23	< 0.001
Labor	11,871 (98.23)	69.27	
**Annual household per capita income**^**c**^			
> 2000RMB	5579 (46.16)	70.84	< 0.001
≤ 2000RMB	5504 (45.54)	67.08	
Unknown	1002 (8.29) ^d^	73.85	
**Marital status**			
Married	11,443 (94.69)	69.69	< 0.001
Unmarried	642 (5.31)	63.71	
**Living arrangement**			
Living with spouse or relatives	11,779 (97.47)	69.73	< 0.001
Living alone	303 (2.51)	55.45	
Unknown	3 (0.02)	100.00	
**Frequency of eating fruits**			
> 3 times/week	5983 (49.51)	71.92	< 0.001
≤ 3 times/week	6102 (50.49)	66.88	
**Frequency of eating vegetables**			
> 3 times/week	11,028 (91.25)	70.29	< 0.001
≤ 3 times/week	1057 (8.75)	59.79	
**Body mass index**			
< 30 kg/m^2^	10,569 (87.46)	70.11	< 0.001
≥ 30 kg/m^2^	1475 (12.21)	64.14	
Unknown	41 (0.34)	68.29	
**Chronic diseases**			
No	10,181 (84.24)	71.81	< 0.001
Yes	1904 (15.76)	56.36	
**Total**	**12,085 (100)**	**69.38**	

^a^ Proportion of respondents reporting no problem in any EQ-5D-3L dimension

^b^*p* values were derived from the Chi-square test on full health

^c^ Annual household per capita income is in RMB

^d^ The 1002 subjects excluded due to missing value on annual household per capita income had better better health status compared to those with no missing values (*p* < 0.001)

The mean (95% CI) for Korean, Chinese (city), US, UK and Japanese health utility scores were 0.963 (0.962–0.965), 0.948 (0.946–0.950), 0.943 (0.941–0.944), 0.930 (0.927–0.932) and 0.921 (0.919–0.924) respectively (Table 2). The difference in health utility scores estimated based on the five tariffs was statistically significant (F = 328.61, *p* <  0.001). The mean difference (95% CI) between the Chinese (city) and four other tariffs were as follows: UK 0.019 (0.018–0.019), US 0.005 (0.005–0.006), Japan 0.027 (0.026–0.027), and Korea − 0.015 -(0.015–0.016) (Supplement Table 1). Differences in scores between any two tariffs were statistically significant (all *p* <  0.0001), but the absolute mean difference did not exceed the MCID.

**Table 2:** The health utility scores derived from the five EQ-5D-3L tariffs in 12,085 residents from rural Hua County, China

**Tariffs **^**a**^	**Mean**	**95% CI**	**SD**^**b**^	**Median**	**IQR **^**c**^	**Min**	**Max**
Korea	0.963	0.962–0.965	0.06	1	0.087	0.151	1
China	0.948	0.946–0.950	0.09	1	0.131	−0.002	1
US	0.943	0.941–0.944	0.09	1	0.173	0.083	1
UK	0.930	0.927–0.932	0.12	1	0.204	−0.349	1
Japan	0.921	0.919–0.924	0.12	1	0.232	0.232	1

^a ^The health utility scores derived from the five tariffs was statistically significant (F = 328.61, *p* < 0.0001)

^b ^Standard deviation

^c ^Interquartile range

As shown in Table 3, any two of these five tariffs had excellent agreement (ICCs> 0.89, 95% CI:0.755–0.969) with the exception of the Korean tariff. The Korean tariff showed poor agreement with the US, UK and Japanese tariffs and the corresponding ICCs were lower than other ICCs [0.860 (95% CI:0.734–0.915); 0.758 (95% CI:0.570–0.848); 0.715 (95% CI:0.451–0.831)].

**Table 3:** The ICCs derived from the five EQ-5D-3L tariffs in 12,085 residents from rural Hua County, China

	**ICC (95%CI)**^**a**^
China/US	0.959 (0.954–0.964)
China/Korea	0.922 (0.836–0.955)
China/UK	0.919 (0.859–0.947)
China/Japan	0.892 (0.755–0.941)
UK/Japan	0.960 (0.953–0.965)
UK/US	0.954 (0.925–0.969)
US/Japan	0.922 (0.838–0.955)
Korea/US	0.860 (0.734–0.915)
Korea/UK	0.758 (0.570–0.848)
Korea/Japan	0.715 (0.451–0.831)

^a^ All *p* values < 0.0001

Bland-Altman plots for each pair among the five tariffs are shown in Supplement Figure 1. Systematic variation was observed with a decreasing trend in the absolute score differences as average health utility scores increased. More than 92% of the differences in individual health utility scores fell within 95% limits of agreement for each pair of the five tariffs, while the Chinese (city) and other tariffs (0.099–0.167) had significantly narrower limits of agreements than the Korean and other tariffs (0.146–0.253). Moreover, we compared the absolute individual score differences of any two tariffs with a MCID of 0.074. 26.97% (3259/12,085), 0.53% (64/12,085), 2.52% (305/12,085), and 4.74% (573/12,085) of the absolute score differences in the Chinese (city) and the Japanese, UK, US, Korean tariffs exceeded the MCID. 30.13% (3641/12,085), 25.73% (3110/12,085), and 18.12% (2190/12,085) of the absolute score differences in the Korean and the Japanese, UK, US tariffs exceeded the MCID, and in almost all these cases, the Korean scores were higher than the Japanese, UK and US scores. In the other pair wise comparisons of two tariffs, the proportions of absolute score differences that exceeded the MCID were less than 10%.

Table 4 shows the discriminative ability of the five tariffs for the factors associated with HRQOL, as listed in Table 1. The EQ-5D-3L scores derived from these five tariffs were significantly different for all the known-groups (*p* <  0.001) except the job type variable, but all of the absolute mean differences by each of the five tariffs were lower than the MCID. The RE statistics of the UK, US and Japanese tariffs were smaller than the Korean and Chinese (city) tariffs in 10 out of 11 variables, and the Chinese (city) tariffs were the most efficient in discriminating the differences in age, job type, living arrangement and BMI variables. The ES statistics of the UK, US and Japanese tariffs were slightly smaller than the Korean and Chinese (city) tariffs in 10 out of 11 variables, indicating the UK, US and Japanese tariffs had lower sensitivity than the Korean and Chinese (city) tariffs. Besides, the ES estimates from the Chinese (city) tariff were the largest in groups related to two variables and the second largest in groups related to nine variables as well.

**Table 4:** Known-groups validity derived from the five EQ-5D-3L tariffs in 12,085 residents from rural Hua County, China

**Variable**^**a**^		**Tariffs**				
		**China**	**UK**	**US**	**Japan**	**Korea**
**Age (years)**						
45–55	Mean (Median)	0.954 (1)	0.936 (1)	0.948 (1)	0.928 (1)	0.968 (1)
56–69	Mean (Median)	0.942 (1)	0.923 (1)	0.938 (1)	0.915 (1)	0.959 (1)
	Difference	0.012	0.013	0.010	0.013	0.009
	Z statistic	6.077	5.519	5.497	5.789	5.639
	Relative efficiency	1.000	0.908	0.905	0.953	0.928
	Effect size	0.130	0.111	0.104	0.112	0.141
**Gender**						
Male	Mean (Median)	0.956 (1)	0.939 (1)	0.951 (1)	0.932 (1)	0.969 (1)
Female	Mean (Median)	0.941 (1)	0.920 (1)	0.935 (1)	0.911 (1)	0.959 (1)
	Difference	0.015	0.019	0.016	0.021	0.010
	Z statistic	8.976	9.382	9.353	9.208	9.747
	Relative efficiency	1.000	1.045	1.042	1.026	1.086
	Effect size	0.170	0.165	0.169	0.174	0.157
**Educational level**						
Middle School and above(>6y)	Mean (Median)	0.954 (1)	0.937 (1)	0.948 (1)	0.929 (1)	0.968 (1)
Primary School and below(≤6y)	Mean (Median)	0.943 (1)	0.923 (1)	0.938 (1)	0.915 (1)	0.960 (1)
	Difference	0.011	0.014	0.010	0.014	0.008
	Z statistic	6.005	5.923	5.889	5.978	6.097
	Relative efficiency	1.000	0.986	0.981	0.996	1.015
	Effect size	0.126	0.115	0.109	0.115	0.128
**Job type**						
Office	Mean (Median)	0.963 (1)	0.947 (1)	0.956 (1)	0.939 (1)	0.975 (1)
Labor	Mean (Median)	0.948 (1)	0.929 (1)	0.942 (1)	0.921 (1)	0.963 (1)
	Difference	0.015 ^b^	0.018 ^c^	0.014 ^d^	0.018 ^e^	0.012 ^f^
	Z statistic	2.208	1.938	1.933	1.967	2.198
	Relative efficiency	1.000	0.878	0.875	0.891	0.995
	Effect size	0.168	0.151	0.151	0.150	0.176
**Annual household per capita income**^**g**^						
> 2000RMB	Mean (Median)	0.953 (1)	0.935 (1)	0.947 (1)	0.927 (1)	0.968 (1)
≤ 2000RMB	Mean (Median)	0.942 (1)	0.923 (1)	0.938 (1)	0.914 (1)	0.959 (1)
	Difference	0.011	0.012	0.009	0.013	0.009
	Z statistic	5.250	4.838	4.825	5.054	5.361
	Relative efficiency	1.000	0.922	0.919	0.963	1.021
	Effect size	0.130	0.104	0.098	0.107	0.138
**Marital status**						
Being married	Mean (Median)	0.949 (1)	0.931 (1)	0.943 (1)	0.922 (1)	0.964 (1)
Unmarried	Mean (Median)	0.931 (1)	0.911 (1)	0.928 (1)	0.902 (1)	0.951 (1)
	Difference	0.018	0.020	0.015	0.020	0.013
	Z statistic	3.824	3.675	3.650	3.654	4.042
	Relative efficiency	1.000	0.961	0.954	0.956	1.057
	Effect size	0.203	0.169	0.165	0.167	0.201
**Living arrangement**^**h**^						
Living only with spouse or relatives	Mean (Median)	0.949 (1)	0.931 (1)	0.943 (1)	0.922 (1)	0.964 (1)
Living alone	Mean (Median)	0.914 (1)	0.889 (1)	0.912 (1)	0.879 (1)	0.940 (1)
	Difference	0.035	0.042	0.031	0.043	0.024
	Z statistic	6.046	5.881	5.894	5.882	5.913
	Relative efficiency	1.000	0.973	0.975	0.973	0.978
	Effect size	0.400	0.351	0.345	0.361	0.382
**Frequency of eating fruits**						
> 3 times/week	Mean (Median)	0.956 (1)	0.938 (1)	0.949 (1)	0.930 (1)	0.970 (1)
≤ 3 times/week	Mean (Median)	0.940 (1)	0.921 (1)	0.937 (1)	0.913 (1)	0.957 (1)
	Difference	0.016	0.017	0.012	0.017	0.013
	Z statistic	7.607	6.979	6.952	7.149	8.227
	Relative efficiency	1.000	0.917	0.914	0.940	1.082
	Effect size	0.183	0.139	0.131	0.147	0.195
**Frequency of eating vegetables**						
> 3 times/week	Mean (Median)	0.950 (1)	0.932 (1)	0.945 (1)	0.924 (1)	0.965 (1)
≤ 3 times/week	Mean (Median)	0.926 (1)	0.904 (1)	0.923 (1)	0.893 (1)	0.947 (1)
	Difference	0.024	0.028	0.022	0.031	0.018
	Z statistic	7.687	7.527	7.525	7.543	8.159
	Relative efficiency	1.000	0.979	0.979	0.981	1.061
	Effect size	0.277	0.235	0.236	0.257	0.280
**Body mass index**^**i**^						
< 30 kg/m2	Mean (Median)	0.950 (1)	0.932 (1)	0.944 (1)	0.924 (1)	0.965 (1)
≥ 30 kg/m2	Mean (Median)	0.935 (1)	0.913 (1)	0.931 (1)	0.905 (1)	0.954 (1)
	Difference	0.015	0.019	0.013	0.019	0.011
	Z statistic	5.377	5.098	5.069	5.219	5.167
	Relative efficiency	1.000	0.948	0.943	0.971	0.961
	Effect size	0.166	0.157	0.148	0.154	0.175
**Chronic diseases**						
No	Mean (Median)	0.954 (1)	0.936 (1)	0.948 (1)	0.929 (1)	0.968 (1)
Yes	Mean (Median)	0.918 (1)	0.893 (1)	0.914 (1)	0.883 (1)	0.941 (1)
	Difference	0.036	0.043	0.034	0.046	0.027
	Z statistic	14.704	14.103	14.095	14.300	14.809
	Relative efficiency	1.000	0.959	0.959	0.973	1.007
	Effect size	0.403	0.372	0.369	0.378	0.413

^a^ All difference scores were statistically significant using the independent t-test except for the job type variable, *p* < 0.001

^b^*p* = 0.0151

^c^*p* = 0.0283

^d^*p* = 0.0285

^e^*p* = 0.0301

^f^*p* = 0.0107

^g^ Annual household per capita income was not available for 1002 (8.29%) subjects, who were excluded from analysis

^h^ Living arrangement was not available for 3 (0.02%) subjects, who were excluded from analysis

^i^ Body mass index was not available for 41 (0.34%) subjects, who were excluded from analysis

Fifty-five of the total 243 possible EQ-5D-3L health states were observed in our study sample. The 55 observed health states resulted in 1485 pairs of health states (C^2^_55_). One-way ANOVA and post-hoc Bonferroni tests showed that the Chinese (city) tariff (0.215) generally led to lower mean absolute transition scores than the Western tariffs (the UK:0.342, the US:0.230) and higher mean absolute transition scores than Asian tariffs (Japan:0.149, Korea:0.189). In 1163 of 1485 (78.32%) pairs of the EQ-5D-3L health states, the five tariffs were consistent in predicting health gain/loss regardless of the magnitude of the gain/loss. The Chinese (city) tariff had the lowest and the highest consistency with the UK (88.66%) and Japanese (93.72%) tariffs. For these consistent health transitions, SRM ranged from 0.79 to 1.08 with the lowest values for the UK and Chinese (city) tariffs. A substantial proportion of differences in transition scores between Chinese (city) and other tariffs were clinically important (81.93%–88.38% difference in transition scores >MCID) (Table 5).

**Table 5:** Absolute transition scores, consistent health transition and standardized response mean for the observed EQ-5D-3L health transitions in 12,085 residents from rural Hua County, China

**Tariffs**	**Absolute mean transition score**	**95% CI**	**Consistent health gain/loss (China vs.) (%)**	**Standardized response mean for consistent transitions (*n*** **= 1163)**	**Clinically important difference in transition scores compared with China (%)**
China	0.215	0.21–0.22	–	1.08	–
UK	0.342	0.33–0.36	88.66	0.79	88.38
US	0.230	0.22–0.24	90.96	0.83	81.93
Japan	0.149	0.14–0.16	93.72	1.04	83.12
Korea	0.189	0.18–0.20	89.68	1.02	85.58

## Discussion

The EQ-5D-3L scale is recommended for use in health services decision making, and for quantifying quality adjusted life years (QALYs) in cost-utility analysis by a number of Health Technology Assessment bodies [2]. In China, it has also been adopted for population health surveys [9, 10], clinical trials [14] and population-level screening studies [22] for over 20 years. However, it was as yet unclear which of the five published tariffs would be the most suitable for application in rural Chinese populations. To our knowledge, this was the first study providing evidence about the performance of the UK, US, Japanese, Korean and Chinese (city) tariffs for the EQ-5D-3L scale in a general rural population in China. Compared to the UK, US, Japanese and Korean tariffs, the Chinese (city) tariff was found to provide a better level of agreement, known-groups validity and sensitivity to reflect the discrepancy between varied levels of HRQOL related factors, and the performance of observed health transitions revealed that the Chinese (city) tariff would lead to moderate changes in QALYs for this rural population.

This study indeed found statistically significant differences in scores between any two of the five tariffs, and this was similar to findings in previous studies [5, 6, 13]. In addition to variations in the valuation protocol and method used for developing varied tariffs, inherent differences in populations may be a potential explanation for the observed differences among different tariffs, including cultural norms, population characteristics, state of health, values, belief, clinical practice and access to health care services, etc. Given that use of different tariffs may yield different utility scores and cost-utility results, evaluation of the performance of these five tariffs among rural populations in China is warranted.

In this study, we assessed the performance of the five tariffs based on level of agreement, known-groups validity and sensitivity for reflecting the discrepancy between varied levels of HRQOL related factors in this rural population. The Korean tariff had the lowest level of agreement with other tariffs compared with the level of agreement among four other tariffs, and was in disagreement with the UK, US and Japanese tariffs regarding what constituted “good” and “bad” health states. Moreover, Bland-Altman plots demonstrated the possibility that the Korean tariff systematically valued same health states higher than other tariffs do. These findings differed from a community-based study in Shenzhen, China, which investigated over 3028 inhabitants aged 15 and older and reported excellent agreement among the UK, Japanese, Korean and Chinese (city) tariffs [13]. Possible explanations for these differences in findings were as follows. First, the proportion of respondents with health problems was as high as 30.62% in our study, in contrast to 7% in the study by Wu et al. [13]. The discrepancy among different tariffs would be smaller for better health status, but larger for severe health status. As a result, the level of agreement between the Korean tariff and other tariffs in this rural population with poor health status would significantly decrease. Second, pain/discomfort, which is the most common problem worldwide with a prevalence ranging from 29.4 to 61.3% [9], had different effect on the health of individuals in different countries, and the effect on health utility scores from the Korean tariff was the smallest compared to four other tariffs. For example, the effect of health state “11,121” valued by the Korean tariff was minimal with a utility value of 0.037, while the effect derived from the UK, US, Japanese and Chinese (city) tariffs was relatively larger with utility values of 0.080 to 0.173. Therefore, the Korean tariff tended to overestimate the actual level of HRQOL in general rural populations in China, thus decreasing its comparability with other tariffs in health utility scores.

With respect to known-groups validity, the absolute mean differences in all known-subgroups of each of the five tariffs were lower than the MCID. This result was different from previous studies, where the absolute mean differences in some known-subgroups exceeded the MCID [6, 13, 23]. The main reason for this difference was as follows. Compared to populations in previous studies covering entire adult stage and different education levels, subjects in our study were mainly limited to farmers who were not well educated aged 45–69 years, and their living conditions had stronger homogeneity, resulting in a lesser quality of life variation in different subgroups. Therefore, the absolute mean differences of these five tariffs in all known-subgroups did not exceed the MCID. Thus, we further observed that the EQ-5D-3L tariffs might have limited discriminative ability in this rural population in China, but the RE and ES statistics from the UK, US and Japanese tariffs were continuously lower in 10 out of 11 variables than the statistics from Korean and Chinese (city) tariffs, which prompted the view that Western and Japanese tariffs might provide lower sensitivity than Korean and Chinese (city) tariffs in this Chinese general rural population. Korea and China are Asian countries sharing similar cultures and values, and the Asian tariffs may thus better reflect health preferences in rural Chinese populations than the Western tariffs do. These findings were similar to previous studies which had also found that the Western tariffs had poorer discriminative ability than the Asian tariffs in Asian populations [13, 23]. The results stressed the importance of using local tariff instead of international tariffs in context-specific decision-making processes. In addition, the Japanese valuation study was undertaken more than 17 years ago [8], while Korean and Chinese (city) valuation studies were conducted in 2009 and 2014 respectively [7, 17]. With socio-economic development, medical practice patterns, access to health services and public health status may change as well, relevant to secular trends in social preferences, which may lead to a difference in health preferences of more than a decade ago as compared with the current people’s understanding of health. This finding shows that health valuation study will be a long-term undertaking that deserves constant attention and timely updating.

The Chinese (city) tariff generally led to smaller changes than Western tariffs and higher changes than two other Asian tariffs in QALYs which translated into less and greater favorable incremental cost-effectiveness ratios (ICERs). In addition, the Chinese (city) tariff had smaller values than the Korean tariff and higher values than the UK, US, and Japanese tariffs. Therefore, compared to the Western tariffs, the Chinese (city) tariff had higher values attached to impaired health states, which resulted in higher utility gain from life-extending interventions and smaller utility gain from quality of life-improving measures. This implied that application of the Chinese (city) tariff in place of Western tariffs would result in more favorable ICERs for life-extending interventions and less favorable ICERs for quality of life-improving interventions. The findings suggested that choice of tariff might have important impact on economic evaluation studies and funding decisions. It has to be reiterated that the choice of which tariff to use in empirical studies should be based on specific study questions and not on how attractive the resulting ICER might be.

Generally speaking, an appropriate tariff should be able to not only reflect the actual level of the quality of life of a population and facilitate comparison with other health-care evaluation programs (external validity), but should also be able to fully demonstrate the differences in various levels of HRQOL related factors in the study population (internal validity). Based on the analysis above, the Chinese (city) tariff was the most suitable of five tariffs under evaluation and showed no obvious weakness, and would be suitable for use when assessing HRQOL using EQ-5D-3L in a rural population in China.

The main limitation of this study was that it was a single center investigation of residents aged 45–69 and individuals with severe conditions were not included. Thus, our results and conclusions should be interpreted with caution if they are being considered for generalization to other populations with broader age ranges and/or individuals with severe medical conditions involving disability or bedridden illness. As a consequence of cross-sectional design of this study and lack of data on changes in health status over time, all possible transitions were treated as if they had the same probability of occurrence. However, some theoretical health states or transitions are less likely to happen in reality.

## Conclusions

This study systematically evaluated the performance of tariffs derived from the UK, US, Japan, Korea and China (city) in a rural Chinese population based on a large randomized controlled trial in rural China. We recommend adopting this Chinese (city) tariff when applying EQ-5D-3L to assess quality of life among the elderly in China’s agricultural region with socio-economic status similar to Hua County. Results of this study had provided a crucial basis for health surveys, health promotion projects, health intervention trials and health economic evaluation taking HRQOL as a target in rural areas of China, and results will also serve as a foundation for cost-utility analysis in the ESECC trial in the future.

## Supplementary information

**Additional file 1: Supplement Table 1.** Descriptive statistics of differences derived from the five EQ-5D-3L tariffs in 12,085 residents from rural Hua County, China.

**Additional file 2: Supplement Figure 1.** Bland-Altman plots of the five EQ-5D-3L tariffs in 12,085 residents from rural Hua County, China.

## Data Availability

The datasets used and analysed during the current study are available from the corresponding author on reasonable request.

## Abbreviations

HRQOL: Health related quality of life
EQ-5D-3L: Three-level European quality of life five-dimension
UK: United Kingdom
US: United States
GDP: Gross Domestic Product
ESECC: Endoscopic Screening for Esophageal Cancer
BMI: Body mass index
ICCs: Intraclass correlations coefficients
MCID: Minimal clinically important difference
RE: Relative efficiency
ES: Effect size
ANOVA: Analysis of variance
SRM: Standardized response mean
QALYs: Quality adjusted life years
ICERs: Incremental cost-effectiveness ratios
