# Supplementary material for: An evaluation of EQ-5D-3L health utility scores using five country-specific tariffs in a rural population aged 45–69 years in Hua county, Henan province, China
Source: Health Qual Life Outcomes. 2020 Jul 13;18:228. doi: 10.1186/s12955-020-01476-z (PMC7359608; doi:10.1186/s12955-020-01476-z)
Supplement: Supplementary file 1 — Additional file 1: Supplement Table 1. Descriptive statistics of differences derived from the five EQ-5D-3L tariffs in 12,085 residents from rural Hua County, China. [file 12955_2020_1476_MOESM1_ESM.docx]

| **Supplement Table 1. Descriptive statistics of differences derived from the five EQ-5D-3L tariffs in 12,085 residents from rural Hua County, China** | | | | | | | |
| --- | --- | --- | --- | --- | --- | --- | --- |
| **Tariffs ^a^** | **Mean** | **95% CI** | **SD ^b^** | **Median** | **IQR ^c^** | **Min** | **Max** |
| China/UK | 0.019 | 0.018-0.019 | 0.038 | 0 | 0.027 | -0.439 | 0.042 |
| China/US | 0.005 | 0.005-0.006 | 0.025 | 0 | 0.000 | -0.240 | 0.107 |
| China/Japan | 0.027 | 0.026-0.027 | 0.043 | 0 | 0.077 | -0.101 | 0.234 |
| China/Korea | -0.015 | -(0.015-0.016) | 0.027 | 0 | 0.044 | -0.054 | 0.174 |
| UK/US | -0.013 | -(0.013-0.014) | 0.029 | 0 | 0.031 | -0.432 | 0.023 |
| UK/Japan | 0.008 | 0.008-0.009 | 0.033 | 0 | 0.028 | -0.581 | 0.085 |
| UK/Korea | -0.034 | -(0.033-0.035) | 0.059 | 0 | 0.075 | -0.536 | 0.011 |
| US/Japan | 0.021 | 0.021-0.022 | 0.037 | 0 | 0.059 | -0.214 | 0.131 |
| US/Korea | -0.021 | -(0.020-0.021) | 0.037 | 0 | 0.046 | -0.286 | 0.04 |
| Japan/Korea | -0.042 | -(0.041-0.043) | 0.064 | 0 | 0.122 | -0.173 | 0.081 |
| ^a^ Comparison of any two tariffs from these five tariffs was statistically significant, *p*<0.0001 | | | | | | | |
| ^b^ Standard deviation | |  |  |  |  |  |  |
| ^c^ Interquartile range | | |  |  |  |  |  |
