# Supplementary material for: An evaluation of EQ-5D-3L health utility scores using five country-specific tariffs in a rural population aged 45–69 years in Hua county, Henan province, China
Source: Health Qual Life Outcomes. 2020 Jul 13;18:228. doi: 10.1186/s12955-020-01476-z (PMC7359608; doi:10.1186/s12955-020-01476-z)
Supplement: Supplementary file 2 — Additional file 2: Supplement Figure 1. Bland-Altman plots of the five EQ-5D-3L tariffs in 12,085 residents from rural Hua County, China. [file 12955_2020_1476_MOESM2_ESM.docx]

**Supplement Figure 1. Bland-Altman plots of the five EQ-5D-3L tariffs in 12,085 residents from rural Hua County, China**
